# Ocular Health of *Octodon degus* as a Clinical Marker for Age-Related and Age-Independent Neurodegeneration

**DOI:** 10.3389/fnint.2021.665467

**Published:** 2021-04-13

**Authors:** Lily Y. -L. Chang, Nicolas Palanca-Castan, David Neira, Adrian G. Palacios, Monica L. Acosta

**Affiliations:** ^1^School of Optometry and Vision Science, Faculty of Medical and Health Sciences, The University of Auckland, Auckland, New Zealand; ^2^Centro Interdisciplinario de Neurociencia de Valparaíso, Universidad de Valparaíso, Santiago, Chile; ^3^New Zealand National Eye Centre, The University of Auckland, Auckland, New Zealand; ^4^Centre for Brain Research, Faculty of Medical and Health Sciences, The University of Auckland, Auckland, New Zealand; ^5^Brain Research New Zealand—Rangahau Roro Aotearoa, Auckland, New Zealand

**Keywords:** retina, Alzheimer’s disease, degus, aging, eye, amyloid protein, pupil

## Abstract

The aging process and age-related diseases such as Alzheimer’s disease (AD), are very heterogeneous and multifactorial, making it challenging to diagnose the disease based solely on genetic, behavioral tests, or clinical history. It is yet to be explained what ophthalmological tests relate specifically to aging and AD. To this end, we have selected the common degu (*Octodon degus*) as a model for aging which develops AD-like signs to conduct ophthalmological screening methods that could be clinical markers of aging and AD. We investigated ocular health using ophthalmoscopy, fundus photography, intraocular pressure (IOP), and pupillary light reflex (PLR). The results showed significant presence of cataracts in adult degus and IOP was also found to increase significantly with advancing age. Age had a significant effect on the maximum pupil constriction but other pupil parameters changed in an age-independent manner (PIPR retention index, resting pupil size, constriction velocity, redilation plateau). We concluded that degus have underlying factors at play that regulate PLR and may be connected to sympathetic, parasympathetic, and melanopsin retinal ganglion cell (ipRGC) deterioration. This study provides the basis for the use of ocular tests as screening methods for the aging process and monitoring of neurodegeneration in non-invasive ways.

## Introduction

Alzheimer’s disease (AD) is a debilitating illness, and the leading cause of dementia worldwide. Although the disease onset can be relatively early in life, especially in familial AD, its prevalence and morbidity are closely tied to the aging process. With population aging worldwide, a dramatic rise in the incidence of AD is imminent. There are over 50 million people worldwide living with dementia in 2020 and this is predicted to increase to 152 million in 2050 (Alzheimer’s Disease International, 2015). The signs, symptoms and progression of AD are very heterogeneous and multifactorial, which makes it challenging to diagnose the disease based solely on genetic, behavioral tests or clinical history (Schneider et al., 2014). Behavioral testing can be unreliable when the patient is uncooperative or simply lacks attention/motivation to complete the task at the time of examination. Diagnostic tests such as positron emission tomography (PET), structural magnetic resonance imaging (sMRI), and cerebrospinal fluid (CSF) analysis have the advantage of being objective measurements that do not require significant patient cooperation. These specialized tests are however less accessible, expensive, and invasive. In the pursuit of an earlier AD diagnosis, it is also questionable whether the detection of amyloid beta (Aβ) plaques and tau biomarkers are specific enough for AD and whether they are only present in established AD cases but not sensitive enough as early diagnostic signs of AD (Klein and Kaye, 2013). An effective screening method is required to identify AD at the very early stages of the disease, so that intervention can be initiated as early as possible to optimize therapeutic success. For example, clinical ocular signs associated with AD are regarded as potential novel diagnostic techniques for earlier diagnosis, both at structural and functional levels (Chang et al., 2014, 2015, 2016, 2019). Investigations into the pathology of AD in the eye have identified reliable molecular biomarkers (Gasparini et al., 2011; Koronyo-Hamaoui et al., 2011; Frost et al., 2013, 2017; Koronyo et al., 2017), which is a supporting evidence that the disease may affect other organs in addition to the brain. The retina is a part of the central nervous system (CNS) that unlike the brain, can be visualized non-invasively because of its accessibility *via* the clear ocular media. Several potential clinical markers have already been described to complement current clinical protocols for the diagnosis and monitoring of AD (Du et al., 2015; Chang et al., 2016). Therefore, screening tests targeting retinal function are being considered for the detection of AD and other neurodegenerative disorders (La Morgia et al., 2017; Meltzer et al., 2017). However, it is yet to be explained what readily-available ophthalmological tests relate specifically to aging and neurodegenerative processes. To this end, we have selected the common degu (*Octodon degus*) as a model for aging which develops AD-like signs to conduct ophthalmological screening methods that could indicate neurodegeneration.

Degus have been widely used in biological and behavioral studies, and a brain atlas of the degus is available in literature (Kumazawa-Manita et al., 2013a,b) with growing interest as an animal model of aging and AD (Ardiles et al., 2013; Hurley et al., 2018). There are more than 200 articles that describe their biology and more than 30 peer-reviewed articles that highlight degus’ importance in neurobiology research. Their full genome has been sequenced by the National Human Genome Institute and reported by the Broad Institute of MIT and Harvard. Degus can live up to 10–12 years in captivity, which makes them ideal for investigating the effects of aging and age-related diseases (Edwards, 2009; Homan et al., 2010; Ardiles et al., 2013). As they age, degus develop signs of inflammation in the brain and in the retina, as well as cognitive deterioration (Van Groen et al., 2011; Inestrosa et al., 2015; Chang et al., 2020). Particularly, older degus have been shown to develop molecular hallmarks of AD such as an increase in Aβ peptides and phosphorylated Tau expression (Inestrosa et al., 2005, 2015; Van Groen et al., 2011; Ardiles et al., 2012, 2013; Tarragon et al., 2013; Du et al., 2015; Szabadfi et al., 2015; Cisternas et al., 2018). The accumulation of AD-related proteins in the inner layers of the retina was also found to be concurrent with the activation of microglial cells (Chang et al., 2020). It was speculated from these findings that the increase in AD-associated protein levels created cellular stress, promoted neuro-inflammation and eventually led to deficits in synaptic communication in the retina and transmission through the optic nerve. It is of importance to investigate whether these molecular changes seen in degus’ eyes are correlated with poor visual function and ocular health. Here, common methods of human ocular health examination were adapted for the assessment of ocular parameters in the degus. These methods are broadly used in identifying and characterizing retinal diseases in humans and in animal models (Chang et al., 2014). Due to degus’ diurnal nature and previous reports of poor pupil light reflex in AD, it was also deemed valuable to investigate degus’ pupil reflex that are modulated by the melanopsin system and the autonomic nervous system (Palanca-Castan et al., 2020). The results from this study provide the basis for the use of non-invasive ocular tests as screening methods for both age-related and age-independent neurodegeneration. Although there is no clear connection between ocular health and AD-like neurodegeneration in degus, we investigate the applicability of non-invasive ophthalmological evaluations for the purposes of early diagnosis and disease monitoring in longitudinal studies.

## Materials and Methods

### Experimental Animals

The *Octodon degus* (common degu) colony is kept at the Breeding Unit of the Universidad de Valparaiso (UV) in Valparaiso, Chile. All experimental procedures followed bioethics protocols approved by Universidad de Valparaiso following international guidelines on animal handling and manipulation and the Chilean National Agency for Research and Development (ANID).

Animals were housed in a temperature and light controlled enclosure at 18–20°C with a daily cycle of 12 h of light (~300 lux) and 12 h of darkness (<60 lux). The colony was maintained in standard housing and breeding conditions (Palacios and Lee, 2013). Male and female degus were housed in separate cages with 3–4 degus per cage. Degus from the same litter or same family were kept in the same cage and returned to the same cage post manipulation or behavioral testing, to avoid aggression due to anxiety and unfamiliarity. The degus had a wheat-based rodent diet (Prolab^®^ RMH 3000) and water *ad libitum* (Palacios and Lee, 2013). Each cage was equipped with a rodent wheel and swing for physical activity.

This investigation used a total of 79 animals ranging from 4 to 110 months of age (Table 1). Data was obtained from two series of experiments and measurements. The first series comprised 48 animals that were examined for cataracts and had their intra ocular pressure (IOP) measured. Twenty-six of these were also subjected to pupillary light reflex (PLR) tests. The second series repeated the IOP and PLR measurements in 26 and 19 other animals, respectively, totaling 74 IOP measurements and 45 PLR measurements. For analysis purposes, degus were first stratified into four age groups: Juvenile (up to #12 months), Young (13–24 months), Adult (25–48 months), and Aged adult (≥49 months old), followed by a separate analysis using age as a continuous variable. Lens opacities were scored based on clinical observations described in Table 2.

**Table 1:** Age and gender of degus that underwent clinical ocular examination.

	Age range	Average age	Sample size	Female:Male
	(months)	(months)		
Juvenile	1–12	7.50 ± 2.99	16	7:9
Young	13–24	18.07 ± 3.63	15	10:5
Adult	25–48	35.00 ± 7.06	22	14:8
Aged Adult	>48	70.81 ± 14.05	26	15:11

**Table 2:** Scoring criteria for lens opacities.

Score	Signs
Score #1	A lens with no opacities.
Score #2	Small opacities affecting less than 25% of the pupillary area without affecting the central visual axis.
Score #3	Opacities affected approximately 25% of the pupillary area and were encroaching onto the visual axis.
Score #4	Opacities affected 50% of the pupillary area and encroached onto the visual axis.

### Clinical Ocular Tests in *Octodon degus*

Clinical ocular tests in degus were carried out at the UV, Chile. The degus were examined *in vivo* by conducting direct ophthalmoscopy, intraocular pressure (IOP) and pupillary light reflex.

#### Direct Ophthalmoscopy

The degus were handled by a designated vet, who was familiar with the animals and held them wearing leather gloves. Degus were allowed to adjust to the researchers and the lab for about 15 min before tests were carried out. A face mask was worn by the investigator for protection from degus dander and dust due to close working distance. The ocular media (cornea and crystalline lens) of the degus was checked for opacities using a direct ophthalmoscope (Welch-Allyn, NY, USA). As the light from the ophthalmoscope was shone into the degu’s eye, a red reflecting light from the retina was observed. Opacity in the media was seen as a shadow in the red reflex and was documented by drawing. The extent of lens opacity was further graded according to Lens Opacities Classification System III (LOCSIII; Chylack et al., 1993).

#### Intraocular Pressure Measurements

An Icare^®^ tonometer (Icare Finland Oy, Vantaa, Finland) was used to measure the IOP in degus. No anesthesia was required as the contact between the probe and cornea is gentle and quick. The Icare^®^ tonometer uses the rebound technology in which a very lightweight probe makes momentary contact with the cornea. The principle of rebound technology is based on the measurement of contact time and deceleration of the probe during contact with the cornea. The deceleration of the probe and other rebound parameters change as a function of IOP; the higher the IOP, the faster the probe rebounds and decelerates. The degus were carefully restrained by the vet, and IOPs were measured in both eyes in relaxed animals. Care was taken that the IOP measurements were taken from the central cornea, as corneal thickness variation due to eccentricity can affect the IOPs obtained (Saenz-Frances et al., 2013). The baseline IOP was measured at the beginning of the eye examination. A group of degus received topical 1% tropicamide (Minims Tropicamide, Bausch and Lomb, NY, USA) to dilate the pupil. The post-pupil dilation IOP was measured approximately 20 min after the instillation of tropicamide. The change in IOP was calculated as %IOP change = [(Post-T-IOP − Baseline IOP)/Baseline IOP)] × 100.

#### Pupillary Light Reflex

To check the integrity of the autonomic nervous system, an infrared (IR) videography set-up was developed to measure PLR and pupil appearance. The protocol recorded the dynamics of pupil response to a controlled light stimulus. The protocol was trialed in guinea pigs (Chang et al., 2017) before applying the procedure to degus. This set-up consists of a laptop computer connected to an IR camera, an LED stimulus generator, and an animal stand. This protocol records the consensual pupil response when the contralateral eye is stimulated by light. Anesthesia was not required. Animals were dark-adapted for at least 1 h before the experiments and handled gently for 10–15 min until they appeared relaxed.

The degus’ left eye was illuminated by an LED light, and the right pupil response was recorded by the IR camera. The procedure was repeated twice per stimulus. The stimulus intensity was calibrated before each series of stimuli using a Newport 1918-R power meter (Newport Corporation, CA, USA). Data was obtained after using white LED light form a smartphone flash light or from a computer-controlled LED array that allowed applying a single wavelength of 450 nm. The experimental stimuli consisted of two series of three 1-s pulses with an intensity of 72 mW/cm^2^. The inter-stimulus interval was 15 s, and animals were allowed to recover for 15 min between the two series. This single wavelength PLR response is the standard procedure at the Universidad de Valparaiso (Palanca-Castan et al., 2020).

PLR recordings were split into frames and the pupil constriction time course was extracted using a custom Matlab script (Mathworks, Natik, MA, USA; Palanca-Castan et al., 2020). From these recordings, frames, maximum constriction and constriction velocity as well as redilation speed and redilation plateau were calculated (Adhikari et al., 2015b). In addition, the post-illumination pupillary response (PIPR), which reflects the activity of intrinsically-photosensitive retinal ganglion cells (Adhikari et al., 2015b), was measured 5 s after the stimulus (referred to as PIPR_5_). As PIPR is necessarily tied at least partially to maximum constriction, we disentangled this overlap by calculating a retention index equivalent to the fraction of the maximum constriction retained at 5 s after stimulus using the formula:

ϕ  =   (constriction(5s)   /   maximum   constriction)   *   100

where ϕ is the normalized value in %.

Finally, animals were classified according to the benchmark values calculated in Palanca-Castan et al. (2020). Animals with maximum constriction (% pupil size) within the benchmark value ± 2 SD were classified as “good performers” (PLR+), while those with maximum constriction above that were classified as “poor performers” (PLR−).

#### Statistical Analysis

Statistical analyses were done using the Statistical Package for the Social Sciences (SPSS, International Business Machines Corporation, NY, USA). The study sample size (*n* = 8 or more per group and assay) is considered to have the statistical power to test the null hypothesis (Anderson and Vingrys, 2001). Due to our relatively large sample size, statistical analyses were performed twice whenever possible: first using the discrete age groups as a nominal factor and then using age in months as a continuous variable. Significant values are indicated with asterisks: * *p* < 0.05; **p < 0.01; ***p < 0.001.

A One-way ANOVA was conducted for the analysis of opacities grading between the different age groups, using a *post-hoc* Tukey’s test to correct for multiple comparisons.

A Shapiro–Wilks normality test was conducted to assess normality of IOP data distribution when using right/left (R/L) eye, treatment (no treatment/tropicamide) and age group (juvenile/young/adult/aged). Among all comparisons, only the combination of right eye/adults/no treatment violated the assumption of normality (Shapiro–Wilks test, *p* = 0.024). When setting age as a continuous variable, a correlation between data and scores was observed for all comparisons.

We constructed a general linear model to explore the effects of age and tropicamide application on IOP for the R/L eyes using age groups and gender as between-subjects variables, and both tropicamide treatment and eye laterality as within-subjects variables. The analysis was repeated, including age as a continuous covariate.

For pupillometry, variables were normalized and expressed as a percentage of resting pupil diameter. One-way ANOVA was performed to determine whether there were significant differences between age groups, as well as between PLR+ and PLR− animals. Multiple comparisons were accounted for *via* a *post hoc* Tukey correction. Linear regressions were performed in order to test for the relationship between the different variables and age expressed as a continuous factor. In each case, the analysis was performed using bootstrapping with 1,000 resamples (Freedman, 1981).

## Results

### High Incidence of Opacities Among Degus Lenses

**Figure 1:**
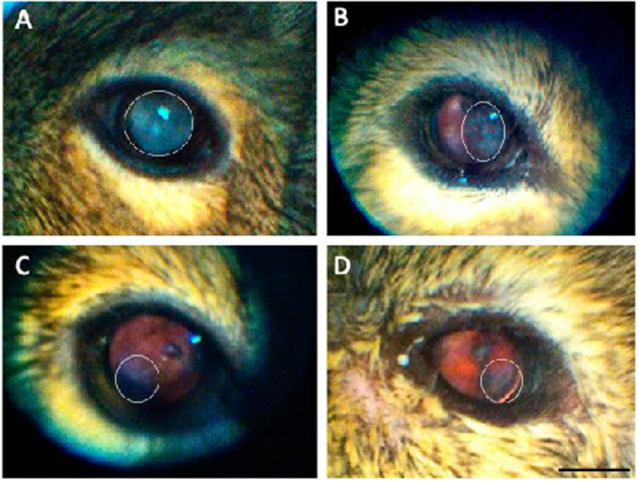
Representative images of the lens opacities seen in degus. **(A)** Servere nuclear cataract in an adult desu. **(B)** Moderate cortical-like cataract in an adult degu. **(C)** Minimal posterior sub capsular-like cataract in an adult degu. **(D)** Mild posterior sub capsular-like cataract in an aged adult degu. White elliptical marquee shows opacity area. Scale bar in **(D)** = 5 mm.

There were no visible signs of opacity in the cornea of animals of any age assessed using direct ophthalmoscopy. The crystalline lens, on the other hand, showed varying degrees of opacities (27/48 animals analyzed: 14 females: 13 males). When opacities were present, they were often observed in both eyes, with asymmetric severity. None of the animals from the Juvenile group had cataracts, while 67% of the Young, 90% of the Adult, and 50% of the Aged adult animals had cataracts. The gender ratios and average cataract score for each age group are presented in Table 3, and representative images of the lens opacities seen in degus are shown in Figure 1.

**Table 3:** Incidence of cataract among female and male degus.

R lens score						
Age group	1	2	3	4	5	Total
1	8	0	0	0	0	8
2	6	7	1	1	3	18
3	1	3	0	0	6	10
4	6	2	1	2	1	12
**Total**	21	12	2	3	10	48

*The cataract score of the right eye was selected for statistical analysis. There was a statistically significant difference between groups as determined by one-way ANOVA (*F*_(3,44)_ = 4.235, *p* = 0.010). A Tukey post-hoc test revealed a significantly higher score in adult degus compared with the other groups*.

### Effect of Age on Intraocular Pressure

The IOP of degus was measured *in vivo* before (baseline IOP) and after (Post-T-IOP) instillation of 1.0% Tropicamide (Minims Tropicamide, Bausch and Lomb, NY, USA) for pupil dilatation. The baseline IOP measures the animal’s normal physiological IOP, while the Post-T-IOP is a measure of any significant IOP spike or drop under the effect of pupil dilatation. Five IOP readings were measured under each condition. Animals were allowed to adjust to the lab and researcher prior to testing, although degus in the Juvenile group were more curious and inquisitive during examination. Table 4 shows the baseline IOP and post-T-IOP values for the different age groups; Supplementary Table 1 shows the IOP values for each degu analyzed.

**Table 4:** Right and left baseline intraocular pressure (IOP; mean ± SD) for each age groups expressed in mmHg.

Age group	Baseline IOP	Baseline IOP	Post-T- IOP	Post-T- IOP	% IOP	% IOP
	(R, mmHg)	(L, mmHg)	(R, mmHg)	(L, mmHg)	change—R	change—L
Juvenile (*n* = 15)	17.87 ± 4.50	18.25 ± 4.37	17.08 ± 3.70	16.81 ± 2.83	−4.42	−7.89
Young (*n* = 15)	18.48 ± 5.73	20.93 ± 5.34	18.47 ± 4.88	19.20 ± 3.47	−0.05	−8.27
Adult (*n* = 21)	19.43 ± 6.40	19.55 ± 6.90	18.77 ± 7.40	19.41 ± 7.1	−3.40	−0.72
Aged adult (*n* = 25)	19.28 ± 3.85	19.06 ± 4.88	24.78 ± 6.40	21.07 ± 3.59	28.53	10.55

*Pre- and post-tropicamide instillation values are shown, as well as the normalized IOP change post-tropicamide*.

Results of the generalized linear model using age groups show no significant effect of sex (*p* = 0.582) or age group on the IOP (*p* = 0.210). A within subjects’ analysis of IOP showed no significant effect of tropicamide (*p* = 0.798) and no significant difference between left and right eyes (*p* = 0.860). However, a significant interaction was found between tropicamide and L/R tested eye (*p* = 0.040*), which was no longer significant when age was treated as a continuous variable. No other significant interactions were found when age groups were treated as a nominal factor. However, when treating age in months as a continuous variable, there was a significant effect of age (*p* = 0.037*) and eye laterality (*p* = 0.030*) as well as an interaction of L/R eye tested vs. age (0.031). All other within-subject factors and their interaction showed no significant effect.

### Pupil Light Reflex

**Figure 2:**
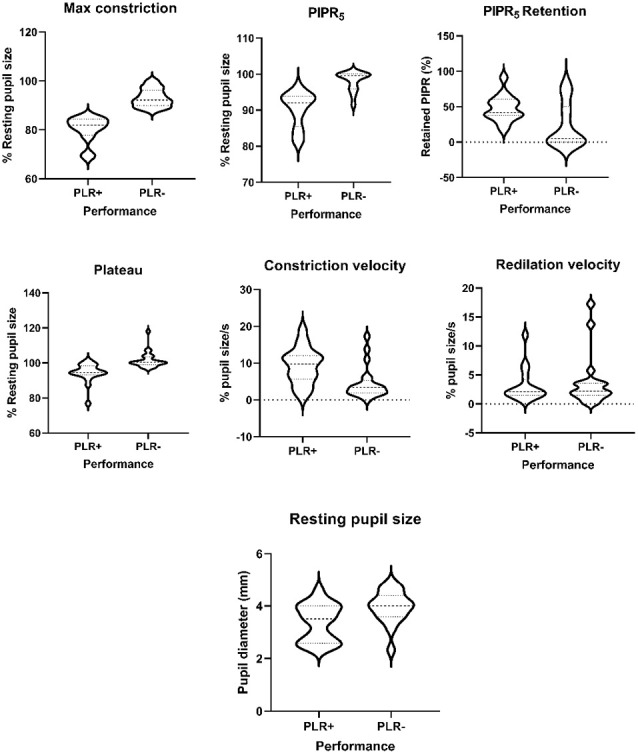
Violin plots of the pupillary light reflex (PLR) parameters in good performers (PLR+) abd poor performers (PLR−) degus.

PLR parameters for the four degu age groups are summarized in Table 5, and a summary of the statistical analysis by age group, or age as a continuous variable are shown in Table 6 and Figure 2. When PLR parameters are analyzed by age groups, one-way ANOVA test showed no statistically significant differences (Table 6). Although the overall result for maximum constriction was significant (*p* = 0.040*), the effect disappeared when we corrected for multiple comparisons. When doing regression analysis with age as a continuous variable, however, both maximum constriction and PIPR_5_ were significantly influenced by the age of the animal (*p* = 0.020* and 0.019*, respectively), where both parameters reduced with advancing age.

**Table 5:** Pupillary light reflex (PLR) parameters analyzed in degus.

	Resting pupil diameter (mm)	Average constricted pupil diameter (mm)	Average dilation velocity (mm/s)	Average redilated pubil diameter (mm)	Average constriction velocity (mm/s)	Average dilation ratio (%)	Average constriction ratio (%)
Juvenile (*n* = 13)	3.46 ± 0.76	3.00 ± 0.83	0.05 ± 0.4	3.40 ± 0.79	0.34 ± 0.11	97.70 ± 2.94	85.33 ± 6.88
Young (*n* = 8)	3.75 ± 0.86	3.28 ± 0.90	0.15 ± 0.23	3.73 ± 0.98	0.29 ± 0.13	98.67 ± 6.67	85.46 ± 8.87
Adult (*n* = 10)	3.70 ± 0.66	3.10 ± 0.93	0.12 ± 0.08	3.7 ± 0.85	0.44 ± 0.18	98.87 ± 7.77	84.64 ± 8.86
Aged adult (*n* = 14)	3.81 ± 0.66	3.18 ± 0.86	0.10 ± 0.15	3.32 ± 1.42	0.47 ± 0.27	99.09 ± 7.12	92.33 ± 5.32

*Abbreviations: J, juvenile; Y, young; A, adult; Aa, aged adult*.

**Table 6:** Summary of the statistical analysis of pupillary light reflex features.

Variable	Between	Effect of age	Between performance
	age groups *p*	(continuous) *p*	groups *p*
Maximum constriction	0.040^*a^	0.004**	<0.001***
Redilation plateau	0.836	0.370	<0.001***
Constriction velocity	0.351	0.271	0.004**
Redilation velocity	0.377	0.306	0.701
PIPR5	0.132	0.019*	<0.001***
PIPR5 retention index	0.589	0.391	0.018*
Absolute resting pupil diameter	0.872	0.423	0.013*

Numbers in bold indicate a significant effect. ^a^indicates an overall significant effect that disappeared when correcting for multiple comparisons. Statistical analysis was One-way ANOVA followed by Tukey’s test multiple comparisons. Significant values are indicated with asterisks: *p < 0.05; **p < 0.01; ***p < 0.001.

On the other hand, when comparing performance groups, the PLR+ group showed significantly smaller pupil resting size (*p* = 0.013), more sensitive responses to light (maximum constriction *p* < 0.001***), higher plateau constriction (*p* < 0.001***), higher constriction speed (*p* = 0.004**) and higher PIPR_5_ (*p* < 0.001***) and relative PIPR retention (*p* = 0.018*). When PLR performance is compared against age, PLR performance and age in months did not show a significant relationship (*p* = 0.082). The PIPR retention index also showed no variation with age (in contrast with maximum constriction and PIPR), whereas it showed large differences between PLR+ and PLR− animals (47.65 ± 18.43% and 24.03 ± 31.23%, respectively). Sex as a factor did not show any significant effect on any of the study variables.

## Discussion

This study provides evidence that degus exhibit age-related changes in selected ocular tissues. Increasing age was found to have a significant effect on the incidence of cataracts, when age was analyzed as a nominal factor (juvenile, young, adult, aged adult groups). When age was treated as a continuous value, IOP was also found to increase significantly with advancing age. As for PLR parameters, even though age had a significant effect on the maximum constriction and PIPR_5_, several parameters were shown to be age-independent (PIPR retention index, resting pupil size, constriction velocity, redilation plateau). Hence, it appears that there are other underlying factors at play that regulates PLR and may be connected to sympathetic, parasympathetic, and ipRGC deterioration. The study represents the first attempt to use common ocular tests as screening tools for the aging process, and monitoring of autonomic and ipRGC function anomalies that may be a sign of underlying neurodegenerative processes.

### Cataract in Degus

This investigation confirmed that there was an age-related increase in the incidence and severity of cataracts in degus. Opacity in the degu lens may be due to aging, or the presence of other systemic health problems such as diabetes or a cumulative effect from multiple factors (Datiles and Fukui, 1989; Ardiles et al., 2013). This was further supported by the fact that some degus developed cataracts at an early age (young group). While the incidence was the highest for the adult age (90% of adult degus tested had cataracts), the occurrence of cataract at ~21 months suggest metabolic problems exist in degus (Ardiles et al., 2013). Although AD-associated cataracts have been reported in human patients (Goldstein et al., 2003), these were described as supranuclear cataracts, seen clinically at the extreme periphery of the crystalline lens. Such cataract morphology was not seen in the degus, making it difficult to draw a correlation between an increased incidence and severity of cataract in degus and in AD.

The cataracts observed in the degus were cortical, posterior-subcapsular, or nuclear, but it was sometimes not possible to discern the cataract subtypes based on red-reflex alone. Future degus lens assessment should include the use of an animal slit lamp biomicroscope for thorough assessment of the crystalline lens. Nevertheless, cataract screening was still considered beneficial to gauge the level of vision the degus had, which would assist in the understanding and analysis of their behavioral testing. For instance, detection of vision problem would explain inability to detect certain stimuli in a behavioral task, which may otherwise be attributed solely to cognitive impairment (Garcia et al., 2010).

### Intraocular Pressure in Degus

The analysis of the IOP measurements using age groups failed to find any effect of age on IOP, although it showed a significant interaction of drugs with eye side. However, this correlation disappeared when age was used as a continuous variable. Instead, there was a significant effect of age and eyesight, both separately and in combination with one another.

Based on the series of statistical analyses in this investigation, it was identified that defining discrete boundaries for age groups can lead to different statistical results. As such, having a large enough sample size allows using age as a continuous variable, not subjected to arbitrary cutoff points, and may provide greater sensitivity in detecting an age-related effect. For example, when age was treated as a continuous variable, there was a significant effect of age, eye laterality and an interaction of both factors with IOP, that was not significant when the analysis used age as a categorical variable. This suggests the presence of an asymmetry in IOP that increases as degus age, and may be associated with more severe anterior chamber narrowing in one eye than the other as observed in aging human eyes (Rufer et al., 2010). However, this speculation is yet to be confirmed, and will require the use of an animal slit lamp biomicroscope.

Given the aforementioned variability in statistical significance, and lack of a confirmed mechanism to explain the differential effect of tropicamide on each eye, we recommend taking this particular finding prudently (Stadtbaumer et al., 2002; Gomes et al., 2011). Previously, it has been reported that application of tropicamide to one eye has an effect on the contralateral eye (Patsiopoulos et al., 2003). In addition, there was high variability in the range of IOP measured in the degus, under no sedation. It could not be ruled out that the variability may have been caused by anxiety and stress. For future studies, degus should be sedated by isofluorane prior to IOP measurements, as recommended by the iLab tonometer manual.

### Pupil Light Reflex in Degus

The results of the PLR analysis comparing animals by age group found no significant effect of age on any of the variables studied (Table 6). However, when using age in months as a continuous variable, age had an effect on both constriction and PIPR_5_. This may be due to the collective impact of additive genetic effects on individuals rather than groups, varying by sex and age; and a reminder that natural animal models have different genetic origins (Muff et al., 2019), and the onset of pathology may be more variable.

The differences in constriction and PIPR_5_ between animals of different ages reflect a general decline in the sensitivity of the animals to light stimuli. The PIPR_5_ is mainly controlled by the melanopsin-containing ipRGCs (Gamlin et al., 2007; Adhikari et al., 2015a; Joyce et al., 2016). However, the maximum constriction and PIPR_5_ were correlated, in that a decrease in maximum constriction implied a decreased PIPR_5_. Therefore, the relative roles of rods, cones and ipRGCs in PIRP_5_ cannot be ascertained with our simplified design, involving either a single white light flash of single blue wavelength rather than two or more wavelengths (Meltzer et al., 2017; Palanca-Castan et al., 2020).

To characterize the pupillary response and disentangle the initial constriction from the PIPR_5_, we calculated a PIPR retention index. The PIPR retention index, unlike the maximum constriction and the PIPR_5_, was not significantly affected by the age of the animal, but was related to the general PLR performance of the animal (see below). Therefore, it is possible that PIPR retention index is more reflective of ipRGC performance than the uncorrected PIPR_5_ which, being tied to maximum constriction, suffers the same nonspecific decline with age.

Although the evaluation of ipRGC function is best done using chromatic pupillometry with two or more relevant wavelengths (e.g., Meltzer et al., 2017), this involves relatively complex setups that are not always available. In such cases, the PIPR retention index could be a useful substitute and a clinical marker of early decline, as specifically ipRGCs have been shown to have increased mortality in AD patients (La Morgia et al., 2017).

The general assessment is that maximum constriction seems to decline with age for many reasons, and with it the PIPR_5_. The retention index, however, is not dependent on age. It is due to performance, and could be used as a clinical marker of ipRGC function. Indeed, comparative analysis of good performers (PLR+) and poor performers (PLR−), showed higher constriction and PIPR_5_ values (or smaller normalized pupil sizes) in PLR+ animals (Table 6). This is not surprising, as they were explicitly classified by maximum constriction, which also influences PIPR_5_. However, constriction plateau, constriction velocity, PIPR retention index and absolute resting pupil size were also significantly different between good performers and bad performers. Notably, these four parameters were not significantly related to the age of the animal, suggesting that variation in the PLR response is influenced by other factors, such as gender and intrinsic physiological pathways discussed in Chang et al. (2017).

The resting pupil diameter reflects the balance between the parasympathetic nervous system (controlling the sphincter pupillae of the iris, causing pupil constriction), and the sympathetic nervous system (controlling the dilator pupillae of the iris, causing pupil dilatation). The relevance of PLR examination in degus is that the sympathetic nervous system was previously reported to be relatively more unopposed due to cholinergic deficiency and a reduced parasympathetic control of the pupil (Fotiou et al., 2009) as shown in preclinical cases of AD in humans (Frost et al., 2017). Therefore, plateau, constriction velocity and resting, non-normalized pupil size are all relevant diagnostic parameters to detect neurological or visual deterioration (Hall and Chilcott, 2018).

### Discussion of the Investigational Methods

Using discrete age groups to test for age-related effects can be problematic, as the cutoff age for groups inevitably have an arbitrary component. As mentioned in the IOP analysis discussion (see above), even a small change can have a significant impact on the analysis results. Specifically, analysis using a continuous measure of age found significant effects on IOP, maximum constriction and PIPR that the age group analysis missed. Moreover, from a data comparability standpoint, different definitions of age group cut-offs in different laboratories can make results less comparable across research groups.

More frequently used transgenic animal models such as inbred colonies of rats or mice are genetically homogeneous, but sporadic animal models such as* O. degus*, can show large degrees of variability (Brekke et al., 2018). This highlights the need for robust analyses and a large sample size in order to discover neurodegenerative effects that would otherwise be easier to identify in more homogenous populations. Collaboration between laboratories that allows for data pooling and standardized analysis protocols would help the development of degus as an animal model for the study of age-related deterioration, as well as other neurodegenerative diseases.

Ophthalmological assessment of degus in this study found possible clinical markers for age-related ocular and neural degeneration (IOP, maximum PLR constriction, PIPR), and age-independent performance (PIPR retention index, resting pupil size, constriction velocity, redilation plateau) that may be connected to sympathetic, parasympathetic, and ipRGC deterioration. However, as the degus assessed in this study were bred and maintained for longitudinal studies, ocular tissue collection and molecular experiments were not possible at the end of clinical assessment.

### Ophthalmological Evaluations and Alzheimer’s Disease

Degus are a sporadic animal model for AD-like neurodegeneration, as several studies have detected molecular and behavioral hallmarks of the disease. Their long lives in captivity also makes them an appropriate animal model for more general age-related neurodegeneration, as demonstrated in numerous investigations to date. The rationale behind utilizing ophthalmological evaluations was to have a screening method for the detection of possible AD cases in our experimental population. However, the results of the ophthalmological evaluations *per se* are not sufficient to detect AD. A limitation of this study was that cognitive assessment, and assessment for systemic diseases was not available for each degu tested for ocular health problems. Therefore, the deterioration of ocular function in our animals may be attributed to aging, age-related neurodegeneration, AD, other systemic diseases or a combination of several of these factors. This limits the conclusion of this study regarding the specific detection of AD.

However, for future studies, *in vivo* ocular examination should be carried out in collaboration with cognitive and behavioral testing (Ardiles et al., 2012) to see if each animal displays signs of cognitive impairment. Concurrent hippocampal electrophysiology and brain immunohistochemistry/western blot data from the same animal would also provide a more complete picture whether AD-specific findings in the brain are associated with specific findings in the eyes, and whether there exist clinical markers that are more directly related to AD rather than other forms of neurodegeneration. Specifically, it would be warranted to test the hypothesis that, as suggested by La Morgia et al. (2017), AD is characterized by a specific decline in ipRGC function. If that was the case, we could expect afflicted animals to show a reduction in PIPR retention, associated anatomical and electrophysiological changes, but not necessarily the more general PLR performance indicators.

## Conclusions

This study, evaluating and comparing cataract incidence, cataract grading, IOP, and PLR parameters in degus, represents the first exploration into using non-invasive ocular screening to monitor age-related changes, and abnormal pupil physiology. In light of the variability across degus cohorts from different labs, this investigation also uncovered the importance of treating age as a continuous variable in data analysis, which provides greater sensitivity to detecting age-related effect, and improves the comparability of data across labs.

Greater cataract incidence, cataract severity, and IOP were identified as viable means of detecting age-related changes in degus’ eyes, while PLR was found to be mostly age-independent and likely to be governed by more complex factors including autonomic nervous system and ipRGC function. Overall, these low-cost, non-invasive ophthalmological assessments were considered promising tools to screen degus for age-related degeneration in the eye, and will play an important role in longitudinal studies, where behavioral testing and molecular experiments are done in conjunction.

The influence of AD on ocular changes remains to be determined. Further investigation including the addition of *in vivo* tests such as retinal imaging using OCT, and electroretinogram are warranted for a more comprehensive evaluation of degus ocular health. These additional tests will also provide evidence on whether AD proteins in the retina are directly causing structural changes in the retina, resulting in clinically detectable deficits in ocular function screening.

## Data Availability Statement

The original contributions presented in the study are included in the article/Supplementary Material, further inquiries can be directed to the corresponding author/s.

## Ethics Statement

The animal study was reviewed and approved by Universidad de Valparaiso following international guidelines on animal handling and manipulation and the Chilean National Agency for Research and Development (ANID) bioethics and biosecurity standards.

## Author Contributions

AP, MA, NP-C, and LC: conceptualization. NP-C, DN and LC: methodology. NP-C and LC: formal analysis. AP and MA: resources. NP-C and LC: data curation. LC and NP-C: writing—original draft preparation. All authors: writing—review and editing. MA and AP: supervision. AP: project administration. MA, AP, and NP-C: funding acquisition. All authors contributed to the article and approved the submitted version.

## Conflict of Interest

The authors declare that the research was conducted in the absence of any commercial or financial relationships that could be construed as a potential conflict of interest.
