# Supplementary material for: Ocular Health of Octodon degus as a Clinical Marker for Age-Related and Age-Independent Neurodegeneration
Source: Front Integr Neurosci. 2021 Apr 13;15:665467. doi: 10.3389/fnint.2021.665467 (PMC8076605; doi:10.3389/fnint.2021.665467)
Supplement: Supplementary file 1 [file Table_1.DOCX]

Supplementary Material

**Table S1:** Right and left IOP for each of our individuals expressed in mmHg. Pre- and post- tropicamide values are shown as well as the normalized IOP change caused by the drug.

| **Degu ID** | **Age (months)** | **IOPR** | **IOPL** | **IOPR (T)** | **IOPL (T)** | **% Change R** | **% Change L** |
| --- | --- | --- | --- | --- | --- | --- | --- |
| 0007ACCA25 | 3 | 17 | 19 |  |  |  |  |
| 471 | 5 | 10 | 10 | 17 | 12 | 70.00 | 20.00 |
| 469 | 5 | 13 | 17 | 10 | 17 | -23.08 | 0.00 |
| 473 | 6 | 17 | 14 | 17 | 15 | 0.00 | 7.14 |
| 452 | 6 | 19 | 20 | 19 | 19 | 0.00 | -5.00 |
| 240 | 6 | 22 | 21 | 17 | 16 | -22.73 | -23.81 |
| 234 | 6 | 26 | 23 | 18 | 19 | -30.77 | -17.39 |
| 229 | 6 | 25.5 | 28.5 | 20 | 21 | -21.57 | -26.32 |
| 227 | 6 | 16 | 17 | 12 | 12 | -25.00 | -29.41 |
| 0007AC673E | 7 | 17 | 18 |  |  |  |  |
| F2EF | 11 | 15.33 | 13.67 | 18.5 | 17.5 | 20.68 | 28.02 |
| 3C19 | 11 | 13 | 14.67 | 14 | 18 | 7.69 | 22.70 |
| C032 | 12 | 16 | 19.67 | 18.5 | 19.5 | 15.63 | -0.86 |
| 2.00E+84 | 12 | 21.25 | 18.25 | 24 | 15.67 | 12.94 | -14.14 |
| 0007ACC43A | 12 | 20 | 20 |  |  |  |  |
| 6794 | 13 | 22.67 | 24 | 24.5 | 24 | 8.07 | 0.00 |
| 170 | 15 | 13 | 20 | 23 | 19 | 76.92 | -5.00 |
| 157 | 15 | 17 | 21 | 18.5 | 18.5 | 8.82 | -11.90 |
| 145 | 16 | 12.5 | 24 | 31 | 23 | 148.00 | -4.17 |
| 143 | 16 | 30 | 33 | 14 | 18 | -53.33 | -45.45 |
| 134 | 16 | 14 | 16 | 20 | 22 | 42.86 | 37.50 |
| 121 | 16 | 12 | 11 | 13 | 15 | 8.33 | 36.36 |
| 106 | 16 | 16 | 19 | 13 | 13 | -18.75 | -31.58 |
| E70C | 17 | 15 | 21 | 16 | 16.5 | 6.67 | -21.43 |
| BFFE | 17 | 15.5 | 16.5 | 20 | 19 | 29.03 | 15.15 |
| BF36 | 22 | 17.5 | 15 | 17 | 14.5 | -2.86 | -3.33 |
| 94 | 22 | 16.5 | 21.5 | 16 | 18 | -3.03 | -16.28 |
| 93 | 22 | 23 | 22 | 16 | 22 | -30.43 | 0.00 |
| 84 | 24 | 24.5 | 28 | 20 | 22 | -18.37 | -21.43 |
| 81 | 24 | 28 | 22 | 15 | 23.5 | -46.43 | 6.82 |
| 174 | 25 | 11 | 15 | 11.5 | 19 | 4.55 | 26.67 |
| 75 | 27 | 21 | 23 | 17 | 21 | -19.05 | -8.70 |
| 61 | 27 | 26 | 29 | 17 | 14 | -34.62 | -51.72 |
| 89 | 28 | 24 | 24 | 26 | 30 | 8.33 | 25.00 |
| 71 | 28 | 27 | 26.5 | 23 | 26 | -14.81 | -1.89 |
| 67 | 28 | 18 | 14 | 22 | 18 | 22.22 | 28.57 |
| 66 | 28 | 27 | 22 | 23 | 21 | -14.81 | -4.55 |
| C043 | 32 | 15 | 12 | 18.3 | 16.66 | 22.00 | 38.83 |
| BD54 | 32 | 24.5 | 20.5 | 20 | 19 | -18.37 | -7.32 |
| D3C7 | 33 | 24 | 31 | 34 | 33.5 | 41.67 | 8.06 |
| BD77 | 35 | 17 | 17 | 17.5 | 21 | 2.94 | 23.53 |
| 19 | 38 | 15 | 10 | 10 | 10 | -33.33 | 0.00 |
| 8 | 40 | 11 | 13 | 23 | 24 | 109.09 | 84.62 |
| 16 | 40 | 19 | 22 | 18 | 27.5 | -5.26 | 25.00 |
| 7 | 41 | 27 | 25 | 33 | 27 | 22.22 | 8.00 |
| 5 | 41 | 9 | 10 | 7 | 6 | -22.22 | -40.00 |
| 330 | 43 | 26 | 24.5 | 18 | 19 | -30.77 | -22.45 |
| 329 | 44 | 10 | 9 | 8 | 7 | -20.00 | -22.22 |
| 323 | 44 | 25 | 27 | 25 | 18 | 0.00 | -33.33 |
| 314 | 44 | 20.5 | 24 | 13 | 15 | -36.59 | -37.50 |
| 304 | 45 | 11 | 12 | 10 | 15 | -9.09 | 25.00 |
| 000796ABCC | 51 | 14 | 14 |  |  |  |  |
| 000796C15A | 52 | 10 | 10 |  |  |  |  |
| 000796D426 | 56 | 23 | 21 |  |  |  |  |
| 000796C7CD | 57 | 21 | 25 |  |  |  |  |
| 000796C49F | 58 | 19 | 15 |  |  |  |  |
| 139 | 61 | 14 | 11 |  |  |  |  |
| 0007ABD5A3 | 61 | 16 | 19 |  |  |  |  |
| 162 | 64 | 22 | 16 |  |  |  |  |
| 000796BECC | 68 | 13 | 15 |  |  |  |  |
| 689 | 70 | 19 | 21 |  |  |  |  |
| 557 | 70 | 23 | 20 |  |  |  |  |
| 82 | 72 | 22.5 | 14.5 |  |  |  |  |
| 671 | 72 | 22 | 19 | 22 | 23 | 0.00 | 21.05 |
| 623 | 72 | 21 | 22 | 27 | 25 | 28.57 | 13.64 |
| 522 | 72 | 22 | 23 | 20 | 18 | -9.09 | -21.74 |
| 300 | 73 | 20 | 19 | 14 | 16 | -30.00 | -15.79 |
| 642 | 74 | 22 | 19 | 27 | 21 | 22.73 | 10.53 |
| 573 | 74 | 19 | 22 |  |  |  |  |
| 000796D49C | 79 | 18 | 21 |  |  |  |  |
| 000796B287 | 80 | 18 | 18 |  |  |  |  |
| 000796C8B5 | 83 | 15 | 15 |  |  |  |  |
| 597 | 84 | 17 | 21 | 32 | 23.67 | 88.24 | 12.71 |
| 377 | 84 | 24 | 32 | 33 | 26 | 37.50 | -18.75 |
| 599 | 96 | 23 | 27 | 29 | 17 | 26.09 | -37.04 |
| 662 | 110 | 24.5 | 17 | 19 | 20 | -22.45 | 17.65 |
